# Family Communication about Diagnostic Genetic Testing for Younger-Onset Dementia

**DOI:** 10.3390/jpm13040621

**Published:** 2023-04-01

**Authors:** Alice Poulton, Lisette Curnow, Dhamidhu Eratne, Adrienne Sexton

**Affiliations:** 1 Department of Paediatrics, The University of Melbourne, Parkville, VIC 3051, Australia; 2 Department of Obstetrics and Gynaecology, The University of Melbourne, Parkville, VIC 3051, Australia; 3 Murdoch Children’s Research Institute, Parkville, VIC 3052, Australia; 4 Monash IVF Group Ltd., Cremorne, VIC 3121, Australia; 5 Victorian Clinical Genetics Services, Royal Children’s Hospital, Parkville, VIC 3010, Australia; 6 Neuropsychiatry, Royal Melbourne Hospital, Parkville, VIC 3051, Australia; 7 Melbourne Neuropsychiarty Centre, The University of Melbourne, Parkville, VIC 3051, Australia; 8 The Florey Institute of Neuroscience and Mental Health, The University of Melbourne, Parkville, VIC 3051, Australia; 9 Walter and Elize Hall Institue of Medical Research, The University of Melbourne, Parkville, VIC 3051, Australia; 10 Genomic Medicine, The Royal Melbourne Hospital, Grattan St, Parkville, VIC 3050, Australia; 11 Department of Medicine-Royal Melbourne Hospital, The University of Melbourne, Parkville, VIC 3052, Australia; 12 Discipline of Genetic Counselling, Graduate School of Health, University of Technology Sydney, Ultimo, NSW 2007, Australia

**Keywords:** dementia, younger onset, communication, genetic counselling, lived experience, genetic testing

## Abstract

Younger-onset dementia (YOD) refers to onset before 65 years of age and may be associated with a genetic cause. Family communication surrounding any genetic risk is complex, and this process may be further complicated in a YOD context due to its effects on cognition, behaviour, and associated psychosocial consequences. This study aimed to investigate how individuals experience family communication about potential genetic risk and testing for YOD. Thematic analysis was performed on verbatim transcripts of nine semi-structured interviews undertaken with family members who attended a neurogenetics clinic due to a relative diagnosed with YOD. The interviews explored the participants’ experiences of learning that YOD might be inherited and the ensuing family communication about genetic testing. Four key themes emerged: (1) a clinical diagnostic odyssey was common and could be a motivator for genomic testing, (2) pre-existing family tension and/or disconnection was a common barrier, (3) family members’ autonomy was considered, and (4) avoidant coping strategies influenced communication. Communication regarding potential YOD genetic risk is a complicated process and may be influenced by pre-existing family dynamics, individual coping mechanisms, and a desire to promote autonomy in relatives. To promote effective risk communication, genetic counsellors should pre-emptively address family tensions that may be exacerbated in the context of genetic testing for YOD, with awareness that family strain during a preceding period of diagnostic odyssey is common. Genetic counsellors can offer psychosocial support to facilitate coping with this tension in an adaptive way. The findings also indicated the importance of extending genetic counselling support to relatives.

## 1. Introduction

Dementia describes a clinical syndrome encompassing cognitive and functional impairment, psychiatric symptoms, and often changes in behaviour [1]. When symptoms of dementia, such as Alzheimer’s and frontotemporal dementia, develop at an early age (before 65 years, considered younger onset dementia), there is a significantly increased likelihood of a monogenic cause [2]. Most single-gene causes of dementia have a dominant pattern of inheritance with high penetrance. Family communication surrounding any genetic risk is often complex and multifaceted, and this communication process may be further complicated in the context of younger-onset dementia (YOD) due to the challenging impact of the clinical presentation of the condition (such as loss of independence, apathy, disinhibition, aggression, and lack of empathy) and the associated psychosocial consequences [3]. Many psychosocial consequences (including changes to social and family relationships, financial and professional status, and mental health) accompany any dementia diagnosis, and these can be amplified in a younger-onset context. Due to the associated behavioural, cognitive, and emotional symptoms, a dementia diagnosis may also lead to changes in lifestyle and intra-family relationships that both probands and caregivers are unlikely to be prepared for [4,5].

Genetic testing can be used in symptomatic YOD patients to identify a specific genetic diagnosis for more accurate prognosis and risk information relevant to biological family members [6,7,8,9]. Family communication about genetics has been studied in other conditions such as familial cancer, but little is known in the context of YOD. Research in other conditions has shown that typically, the responsibility to communicate genetic risks to family members falls to the proband [10]. However, due to the deterioration of cognitive abilities in YOD, the proband may be incapable of making informed independent decisions or explaining genetic test options or results to others. Consequently, family carers may be left with the responsibility of disseminating this information to relevant family members [11]. 

Studies in other genetic conditions have found that the decision of whether or not to disclose genetic information to family members is influenced by several elements, including disease factors, genetic factors, personal issues, family dynamics, and sociocultural influences [3,12,13,14,15,16]. Common motives for disclosure within a family include a perceived obligation to share, an intimate relationship with the family member, and the desire for support. Reasons for nondisclosure include the desire to shield family members from distressing information, the absence of close family relationships, the belief that the family member does not need to know, and the presumption that the information has already been shared by other family members [14]

Typically, communication with close family members occurs quickly, and late disclosure is uncommon [17,18,19,20,21]. Forrest et al. (2003) described the process of genetic information communication as falling into two broad categories: ‘pragmatism’ and ‘prevarication’ [13]. Pragmatism refers to the active and practical disclosure of information, while prevarication refers to a passive and opportunistic approach. When both communication styles are represented in the same family, tensions may arise. There are also different approaches to the process of genetic risk communication. While some individuals are completely open, informing family members from the outset, others employ a more cautious method, limiting disclosure by deliberately downplaying participation in testing and associated genetic risks [18].

Intermediaries may be used in circumstances where the information-giver does not feel comfortable contacting particular family members [20]. The use of intermediaries is particularly evident during the vertical transmission of information [22]. This cascade of responsibility can lead to nondisclosure, as assumptions may be made that at-risk family members have been informed by other family members [23]. In some circumstances, it has been recognized that communication surrounding genetic risk, or lack thereof, can alter family relationships [10,18,23,24]. 

Ultimately, family communication surrounding any genetic risk is often complex due to family dynamics, an incomplete understanding of genetics, and a reluctance to share potentially distressing information [3]. While it is anticipated that the psychosocial implications of a dementia diagnosis will further complicate the process of disclosure in a YOD context, to our knowledge, the family communication that does or does not occur surrounding YOD has not yet been studied. Furthermore, there is limited research examining family communication earlier in the diagnostic pathway—prior to genetic testing. It is important to address this existing gap in research by exploring individual experiences of family communication about genetic risk and/or genetic testing for YOD to inform future counselling practice so probands and family members can be adequately supported throughout the communication process.

This study aims to investigate how individuals experience family communication about genetic risk for YOD with the following key objectives: (i) To describe whether, how, and when genetic risk information regarding YOD is communicated in the family; (ii) To identify what factors facilitate and hinder the occurrence and success of such family communication; (iii) To identify what type of support may be beneficial for individuals communicating genetic risk information regarding YOD in the family.

## 2. Materials and Methods

Due to the investigative nature of the study, a qualitative semi-structured interview design was used [25]. The COREQ checklist for the rigorous reporting of qualitative research findings was applied [26]. A descriptive (general) phenomenological approach was utilised as the theoretical framework of this study. This approach focuses on subjective lived experiences in the context of the lifeworld which the person is interacting with and requires an open-minded reflective stance from researchers [27]. This was appropriate for the given research question, as it facilitated the in-depth exploration of experiences from the perspective of the experiencing person [28]. A purposive sampling method was used to recruit potential participants, facilitating the sampling of a homogenous population with respect to the phenomenological context and enabling the collection of rich, in-depth qualitative data relevant to the topic, with a small sample size appropriate to the qualitative design [29,30].

Eligible participants were those who had attended an appointment at the Neurogenetics Clinic, Royal Melbourne Hospital, within the past 5 years, were over 18 years of age, and resided in Australia. The criterion for being offered a genetic counselling appointment was having a diagnosis of dementia under the age of 65. Genetic testing was an option for people with a diagnosis of dementia under 65 due to the possibility of de novo variants in dominant genes and as some genes have a highly variable age of onset. The clinic covered the cost of genetic testing except for Alzheimer’s disease with onset at over 50 years of age when there was no family history of young onset dementia. During the clinic appointment with a genetic counsellor, the standard approach included an exploration of the motivations for genetic testing, experience of dementia, family history, psychological factors, and the benefits, limitations, and potential impacts of the access of genetic testing with the clients. The family would have the option of more than one pre-test appointment and would meet with the counsellor again to receive the genetic results.

For this research study, due to concerns surrounding the capacity to provide fully informed consent, individuals who had previously received a dementia diagnosis themselves were ineligible to participate. Individuals at risk of Huntington’s disease were also excluded, as the specific genetic implications and higher level of community awareness surrounding Huntington’s disease may have confounded the results.

Thirty potential participants were identified through the Royal Melbourne Hospital Neurogenetics Clinic’s patient database as family members who had attended an appointment regarding YOD in the family. All potential participants received written information regarding the study—a participant information and consent form, as well as a study invitation letter. Signed consent forms were obtained from each participant, and a secondary consent process occurred before each interview, where the investigator verbally explained the study and answered any questions from the participant.

A semi-structured interview guide was used to guide the discussion. Literature on family communication regarding genetic risk and the clinical experience of the study investigators were used to inform the development of the interview guide (Supplementary Table S1). Questions were brief, open-ended, and neutrally framed, and were related to family structure and the timing, content, and process of communication regarding genetic risk between family members. Face-to-face and telephone interviews took place between May and July 2019 and were conducted by student-researcher AP. Interviews were recorded using a dictaphone and were subsequently transcribed verbatim and de-identified (replacing names with pseudonyms and deleting any potentially identifying details such as place names or family member names). Transcribed files were imported to NVivo version 12.0 (QSR International Pty Ltd., Burlington, MA, USA, 2018) for storage, data organisation, and management.

To extract themes from participant responses to open-ended questions, thematic analysis was adopted. This form of analysis, based in a descriptive phenomenological approach, facilitated the identification, examination, and summary of themes within the data, enabling data to be reported in extensive detail with minimal organisation [27]. Coding was a reflexive process and the researchers maintained awareness of their own perspectives in order to minimize bias related to their backgrounds: AS and LC are genetic counsellors with 18 and 12 years of experience in neurogenetics and in conducting and supervising qualitative studies, DE is a consultant neuropsychiatrist with 5 years of clinical experience and a research fellowship in the clinical application of genomics to neurological conditions, and AP is a Master of Genetic Counselling student with training in sensitive interview techniques and qualitative research.

AP reviewed and critically analysed each transcript, identifying and documenting concepts emerging in the data to generate initial codes. A selection of transcripts was independently reviewed and coded by AS and LC, allowing for the comparison and contrast of codes between transcripts, ensuring internal validity. A coding scheme was induced by collating and organising codes into broader hierarchical categories. These categories were used to develop an initial concept map, which facilitated the identification of key themes. Data checking by participants and data saturation were not relevant for this thematic analysis, as the intention was to obtain rich qualitative data.

## 3. Results

All data are presented using pseudonyms, and all other identifying information has been removed to protect the confidentiality of participants. Nine interviews were conducted, and interview lengths varied from 30 to 60 min (Table 1). Seven interviews were conducted by phone and two in person, and there were no differences noted between telephone or in-person interviews. One participant had a family member present for the interview, who also provided consent to participate in this study and was interviewed concurrently. This resulted in a total of ten participants and a 33% (10/30) response rate.

All participants reported an attempt to communicate genetic risk information to their family members. These conversations took place both in person and over the phone, depending on geographical proximity. In this cohort, concerns surrounding emotional burden did not prevent communication with immediate adult family members. Most participants communicated genetic risk with immediate adult family members before accessing testing for the proband. Often, these conversations were prompted by initial genetic counselling appointments. In many circumstances, communication with children, adult family members who were non-receptive to initial genetic risk conversations, and extended family was conditional depending on the outcome of genetic testing, with conversations only being initiated if evidence of a genetic component was identified. Most participants had very clear memories when recounting how YOD was first suspected in their family member. Conversely, memories surrounding when the possibility of a genetic component was first raised and the genetic counselling and testing process were often vague.

Four key themes are presented in the following section. Data in the form of verbatim quotes are used to illustrate these themes.

A clinical diagnostic odyssey is common and can be a motivator for genetic testing, creating an important context for discussions surrounding genetic risk.

In telling their story of how they came to consider a genetic cause for YOD in their family, most participants described a period of time between when symptoms of YOD were first noticed and when a diagnosis was finally made. A difficult journey to diagnosis, commonly known as a diagnostic odyssey—a period of worry, multiple appointments, and misdiagnoses—was labelled by participants as uncertain, frustrating, and distressing.

“Mum was starting to suspect that she may have some sort of dementia, and I was starting to suspect that as well, so when I got back I could see that she was much, much worse, so we went through the process of trying to get some answers. And that was very frustrating and very difficult.”—Jordan

In many cases, misdiagnoses further extended the diagnostic odyssey, which was both exasperating and confusing for participants.

“It took a long time to get diagnosed… [she] had a whole lot of antidepressants first.”—Taylor

“She had one of the neurologists or doctors who was involved from quite early on, and he was actually one of the neurologists who was somewhat in denial and pretty much saying it was only depression.”—Jordan

Furthermore, the difficult experience of a lack of clear diagnosis for a long time prior to the introduction of the idea that there may be a potential genetic component contributing to the YOD motivated some individuals to seek genetic answers.

“I guess in some ways it felt a bit like…the process of genetic testing was to try and find an answer as to what was wrong with [my sister].”—Ashley

“I was hoping for a negative result… but I was also hoping for some form of information.”—Alex

Although it was distressing for family members to learn of a formal dementia diagnosis, many also felt a sense of relief when a diagnosis was finally made.

“I think because we went down that sort of track it wasn’t clear that she had dementia until we were a long way down. And so when she got the diagnosis, she was so deteriorated that it was almost a bit of a relief to know what it was.”—Ashley

“He was aware something was going on. He was quite open about that fact…but it was the fact we didn’t have a formal diagnosis.”—Morgan

“I would love it if there was a test that we could do that could categorically say from day one this is your problem, and this isn’t.”—Blake

For many individuals, this period of uncertainty contributed to family tension, which later set the context in which discussion surrounding genetics occurred.

“I guess I sort of picked up on things that other people in my family were explaining away…to me that was the most exhausting part of this process. It was actually getting her husband and my parents to see that [dementia] was happening”—Ashley

“We had a couple of doctors we saw who were still convinced that [my mum] was just suffering from depression and my dad was being very controlling.”—Jordan

Pre-existing family tension and/or disconnection is a common barrier.

Many participants reported family tension prior to any communication regarding potential genetic risk. Often this tension stemmed from disagreements surrounding the care of a YOD-affected family member.

“We became estranged …mainly due to the treatment of her mother, mainly towards the end. And we’ve had no contact since.”—Jesse

“Dad’s illness placed a lot of strain on all of us. Um…and [my brother’s] illness and behaviour around that placed an enormous amount of strain on all of us.”—Blake

In some circumstances, this tension arose due to behaviour of the YOD-affected family member before a formal diagnosis was reached.

“He was emotionally unavailable... And whether that was part of his own psychiatric illness, or psychological challenges or whether this is part of the spectrum of it... the behavioural variant of frontotemporal dementia... again remains uncertain.”—Blake

Additionally, in some cases, geographical distance also contributed to this disconnect.

“At the time, I wasn’t even in the country… I didn’t return back into Australia until two months after she was diagnosed, actually.”—Sasha

“I suppose we weren’t all together. That’s the thing. We’re not… geographically we’re not all together.”—Leslie

For some families, tension existed prior to any knowledge of YOD in the family.

“We have a lot of differences. Just personality-based differences that we don’t exactly see eye to eye on everything, so we don’t go out of our way to spend time together.”—Sasha

“[There] was always…a little bit of tension there that they do things better than everybody else.”—Alex

“My brother has been estranged from all of our family for 5 or 6 years.”—Morgan

Promoting autonomy of family members is considered.

Many participants reflected on the concept of providing information for relatives when considering whether to communicate genetic risk information to their family members. In many cases, participants were motivated to communicate this information to indirectly facilitate informed, autonomous decision-making for the next generation.

“We wanted to leave as much genetic information as we could down the track.”- Jules

“My two offspring had not had children at that stage, so I thought, well, they can modify their behaviour according to that.”—Jesse

“[I said to my offspring] it’s probably best in your own interests…for your own interests that you actually get this done and clear yourself in any way. I think that’s the sort of stuff you’d want to know straight away…personally…”—Alex

“That was when we went back the second time, and it was more about finding out information for the girls.”—Jordan

“I gave [my offspring] the information that I had available to me, in a way that they could understand.”—Jesse

One participant knew that the results may alter the immediate reproductive decision-making of their sibling, which motivated the decision to access testing:

“[My sister] was also pregnant at the time I was getting testing. Which is one of the reasons we wanted to consider testing.”—Morgan

Another participant emphasised the importance of communicating genetic risk to family members before proceeding with testing for the proband so family members can make informed, autonomous decisions regarding obtaining genetic risk information.

“I just feel like it needs to be a bit more inclusive... so I could choose to be involved, rather than my parents choosing for me.”—Ashley

Avoidant coping strategies influence communication.

When discussing barriers to the communication of genetic risk information, many participants described their family members as adopting avoidance as a coping strategy.

“We probably sound like a very dysfunctional family, but no I never had… I had never really talked to my siblings about…about getting it…we just don’t talk about it. Again, it’s the elephant in the room I guess.”—Sasha

“We had this really intense hour or so with [the genetic counsellor] and then mum goes oh why don’t we go to the [tourist location]. And I said…no... I am absolutely not going [there]. She was just not wanting to talk about it. And that was part of the problem.”—Ashley

“Something that my family is very good at is not having direct conversations like these.“—Ashley

“[My family] put their blinkers on and they don’t really want to know.”—Sasha

Some participants had tried to initiate conversations about genetics and dementia, but relatives did not want to engage.

“[My sister] was very much of the opinion that not knowing is the better fit, and that’s how she could keep it out of mind, and that kind of thing? So she was just very uninterested. So I brought it up once or twice, and then was like…ah okay.”—Morgan

“[My mother] was just not wanting to talk about it. And that was part of the problem.”—Ashley

Intention to communicate about genetics to extended relatives was conditional on whether a definite cause was found:

“We just didn’t have enough information to raise what is a fairly distressing possibility that without further information. I didn’t think it would be helpful.”—Blake

“I was only going to deal with [discussing genetic testing] if it came back positive.”—Morgan

## 4. Discussion

This qualitative study is the first to explore experiences of family communication surrounding YOD genetic risk and testing. This study also contributes to the currently limited research regarding pre-test family communication.

Many participants described existing family tension and disconnection prior to communication regarding genetic risk. Multiple studies have identified dementia as a family divider [31,32,33]. As seen in this cohort, changes in the behaviour and personality of a YOD-affected individual can create conflict and strain [34]. Tension may also arise due to perceived inequalities in the level of caregiving and disagreement regarding what is in the best interest of the affected family member [33]. These pre-existing family dynamics can heavily influence decisions surrounding genetic risk communication in studies on other conditions [13,35]. While nondisclosure was not reported in this sample, family tension was understood to hinder open and effective communication. In some cases, geographical distance also contributed to family disconnect, acting as a barrier to inclusion in conversations regarding potential genetic risk [36]. 

The concept of autonomy emerged when participants recounted their motivations for communicating potential genetic risk. This included a perceived responsibility to make genetic risk information available for the next generation and to facilitate reproductive decision-making. These findings are consistent with existing evidence regarding other conditions, that individuals feel a sense of obligation to share genetic risk information to promote family member autonomy—in particular, the autonomy of younger family members and those approaching reproductive age [6,18,24].

Some participants described the adoption of avoidant coping strategies by their family members as an obstacle to open and effective communication surrounding genetic risk. Lending from the family systems theory, stressors can disrupt the equilibrium of the family emotional unit [37]. Coping strategies can be used to push the family towards a new equilibrium. However, when these coping strategies are incongruent between family members, they may hinder positive adaption [38]. Avoidance is a common feature of coping when individuals are confronted with a threat they perceive as uncontrollable [39,40]. Although avoidance can be an effective short-term mechanism, an overreliance on this strategy in the context of a long-term stressor can be maladaptive [41]. 

In participants’ stories about family communication about genetic testing for YOD, they provided important context about the preceding period where symptoms began, gradually worsened, and there was uncertainty about a diagnosis. Consistent with the literature, many participants described a prolonged period between YOD symptom onset and diagnosis. In Australia, symptoms of YOD are reportedly recognised by family members an average of 3.2 years before clinical diagnosis [42]. This timeline is likely to be further prolonged in a YOD context as family members and healthcare professionals may be unaware that dementia can affect younger people, leading to the dismissal of symptoms, inadequate investigation, or misdiagnosis [43,44]. Other studies have also shown that this period may lead to feelings of frustration and uncertainty [32]. This was the context in which the tensions, motivations, and differing coping strategies relating to communicating about genetics arose.

## 5. Implications

To help facilitate open and productive communication surrounding genetic risk, genetic health professionals should encourage patients to consider the additional strain that may emerge during the communication of genetic risk and should raise awareness of the different coping strategies, including avoidance, which individuals may utilise when learning about potential genetic risk. Furthermore, adaptive coping strategies can be supported, such as positive reinterpretation and growth, through peer support programs. Additionally, given the reportedly indistinct memories of the genetic testing process, genetic health professionals should also consider providing additional resources to aid accurate genetic risk communication, such as written information and online resources, at various stages before and after genetic testing. These efforts, in turn, may reduce the chance of creating further family conflict through reducing the chances of misunderstandings, dissatisfaction regarding selective disclosure, and frustration due to secrecy surrounding genetic risk [10,18,23,24]. Findings also indicate the importance of extending genetic counselling support to family members, particularly those who are younger and those approaching reproductive age.

## 6. Limitations and Future Directions

While the purposive sampling method enabled the identification of information-rich participants, this recruitment method may be biased by willingness to participate. The inclusion criteria were broad and did not focus on families with a strong family history of dementia. Family history may alter genetic risk perceptions and subsequent communication patterns. Additionally, as recruitment was via a genetic service, individuals who participated in the study may be more inclined to participate in conversations regarding genetic risk information compared to those who do not access genetic counselling in this context [13]. All participants were English-speaking, and a proportion had a medical or science background, which may further confound the results. As a retrospective study, participant recollections may have been altered by subsequent experiences and emotions. Consequently, study findings might not represent the actual experience of family communication surrounding the genetic risk of YOD, but rather the remembered experience [18]. Due to the qualitative nature of the study, views of sub-groups (such as those whose relatives’ results showed either a definite genetic diagnosis, a variant of uncertain significance, or no genetic variants) were not compared. Future studies could seek to further explore communication in the settings of these differing results and from the perspectives of specific relatives.

The findings of this study could be used to inform the development of quantitative instruments, such as a questionnaires, to explore this topic further in a larger sample. While this study provides valuable data on experiences of family communication regarding YOD genetic risk, further research is needed to explore these experiences in a sample of individuals who did not access genetic counselling and/or testing. Further research could also investigate family communication later in the diagnostic pathway, after testing has occurred. While some participants were members of the same family, as this was not a purposeful recruitment, responses between members of the same family were not directly compared. Future research could extend the presented findings by purposefully recruiting multiple members of the same family to explore and compare broader family narratives and tensions. The influence of individual perceived inherited risk on intention to communicate with relatives about dementia genetic testing decisions warrants investigation.

## 7. Conclusions

This study explored how individuals experience family communication about genetic risk for younger-onset dementia using semi-structured interviews. Communication regarding potential YOD genetic risk is a complicated process and may be influenced by pre-existing family dynamics, individual coping mechanisms and a desire to promote autonomy in relatives; to promote productive risk communication, genetic health professionals should pre-emptively address family tensions that may emerge in a YOD context and offer psychosocial support to facilitate coping with this tension in an adaptive way. Findings also indicated the importance of extending genetic counselling support to family members.

## Figures and Tables

**Table 1:** Participant demographic information.

Participant Pseudonym *	Age Range	Relationship to Individual Diagnosed with Younger-Onset Dementia	Diagnosis	Genetic Testing Pursued for Proband (Yes/No). If Yes, Outcome of Testing	More than One Relative Diagnosed with Dementia within the Family (Yes/No)
Jules	40–59	Partner	Alzheimer’s disease	Yes, variant of uncertain significance	No
Taylor	60–79	Parent	Alzheimer’s disease	Yes, variant of uncertain significance	No
Morgan	20–39	Offspring	Alzheimer’s disease	No	No
Ashley	40–59	Sibling	Alzheimer’s disease	Yes, variant of uncertain significance	No
Blake	40–59	Sibling	Frontotemporal dementia	Yes, no clinically significant variants identified	No
Jesse	60–79	Partner	Alzheimer’s disease	No	Yes
Jordan	40–59	Offspring	Alzheimer’s disease	No	No
Leslie	40–59	Sibling	Alzheimer’s disease	Yes, variant of uncertain significance	No
Sasha	20–39	Offspring	Younger-Onset Dementia, unspecified type	Yes, variant of uncertain significance	Yes
Alex	60–79	Partner	Frontotemporal dementia	Yes, no clinically significant variants identified	Yes

* Gender-neutral pseudonyms were used to further deidentify participants. Please note, four participants reported having a scientific background. Additionally, four participants were from the same family. This information has not been listed in the demographic table in the interests of participant anonymity.

## Data Availability

The data presented in this study are available on request from the corresponding author.

## Supplementary Materials

The following supporting information can be downloaded at: https://www.mdpi.com/article/10.3390/jpm13040621/s1, Table S1: Interview Guide.
