# Supplementary material for: Family Communication about Diagnostic Genetic Testing for Younger-Onset Dementia
Source: J Pers Med. 2023 Apr 1;13(4):621. doi: 10.3390/jpm13040621 (PMC10144767; doi:10.3390/jpm13040621)
Supplement: Supplementary file 1 [file jpm-13-00621-s001.zip › jpm-2259787-supplementary.pdf]

Supplementary Table S1. Interview Guide

#	Question
1.	I understand you have a family member who was diagnosed with dementia while under the age of 65, which is considered to be a young age. Can you tell me a little bit about how this first came to light?
2.	Do you remember at what point you learnt that this dementia may be inherited?
3.	Some families are in better contact with each other than other families – what is it like in your family?
4.	When the diagnosis of dementia was first raised, did you have any conversations about it in your family?
5.	What were these conversations like? Who was in them? Where did they take place?
6.	Is there anyone you chose not to speak to?
7.	I understand you attended an appointment at genetics at [Hopistal name] with [name of relative with dementia]. What did you expect going into that appointment?
8.	Can you tell me a little bit about what you remember happening at that appointment?
9.	What did you feel you took away from that appointment?
10.	What did you understand about the genetic testing that was offered?
11.	How was the decision to undergo or not genetic testing reached?
12.	Did you think about genetic testing for yourself? What was that process like?
13.	How long did you wait to get results of this testing? How did you cope with waiting?
14.	I understand you attended a result appointment at [Hospital name] with [name of relative with dementia]. Can you tell me what you took away from that appointment?
15.	When you think back on the conversations you had over this period of time, is there anything you would have liked to have done differently?
16.	Do you think there is a different way that genetics services could support you to have these conversations?
17.	Is there anything we haven’t spoken about that you think is important?
